# Aberrant expression of microRNA-132-3p and microRNA-146a-5p in Parkinson’s disease patients

**DOI:** 10.1515/biol-2020-0060

**Published:** 2020-09-02

**Authors:** Yu Shu, Jinjun Qian, Chunyan Wang

**Affiliations:** Neurology Department, The Fourth Affiliated People’s Hospital of Jiangsu University, Zhenjiang 212000, Jiangsu, China; Clinical Medicine, Jiangsu University, Zhenjiang 212013, Jiangsu, China

**Keywords:** Parkinson’s disease, biomarker, diagnosis, miRNAs

## Abstract

Parkinson’s disease (PD) is an age-related neurodegenerative disorder which is assessed based on the motor symptoms. A number of microRNAs (miRNAs) are dysregulated and involved in the pathogenesis or development of PD. However, no confirmed markers are used for the early detection of PD. The present study aimed to elucidate the potential two miRNAs (miR-132-3p and miR-146-5p) as novel markers for early PD diagnosis. In the present study, the expression levels of miR-132-3p and miR-146-5p in serum samples from 82 patients with PD and 44 healthy volunteers were measured by reverse transcription-quantitative polymerase chain reaction. Furthermore, the correlation analysis was performed between aberrant miRNAs and Braak staging, Part V of the Unified Parkinson’s Disease Rating Scale (UPDRS-V; the modified Hoehn and Yahr staging of PD) and Part III of the UPDRS-III. Subsequently, the receiver–operating characteristic (ROC) curve results of miR-132-3p and miR-146-5p from healthy volunteers for PD prediction and from severe PD patients were assessed. From the results it was observed that miR-132-3p and miR-146a-5p expressions were significantly decreased in the serum samples of patients with PD compared to those in the healthy volunteers. Moreover, the expressions of miR-132-3p and miR-146a-5p showed a dramatic decrease in severe PD patients as compared to the normal PD patients. Meanwhile, miR-132-3p and miR-146-5p expressions were negatively correlated with Braak staging (*r* = −0.45, *P* < 0.0001; *r* = −0.51, *P* < 0.0001), UPDRS-III (*r* = −0.55, *P* < 0.0001; *r* = −0.51, *P* < 0.0001) and UPDRS-V scores (*r* = − 0.46, *P* < 0.0001; *r* = −0.45, *P* < 0.0001) in PD patients. The area under the curve (AUC) results of miR-132-3p and miR-146a-5p in discriminating PD patients from the healthy controls were 0.7325 (95% CI = 0.6400–0.8251) and 0.7295 (95% CI = 0.3658–0.8232). Moreover, the AUC results of miR-132-3p and miR-146-5p concerning discriminating severe PD patients from normal PD patients were 0.8175 (95% CI = 0.7229–0.9121) and 0.7921 (95% CI = 0.6937–0.8905). In other words, both miR-132-3p and miR-146a-5p may function as promising biomarkers for early diagnosis of PD.

## 1 Introduction

Parkinson’s disease (PD), characterized by the loss of dopaminergic neurons in the substantia nigra, is the second most common age-related neurodegenerative disorder [1]. To date, dopamine replacement and pharmacological treatments were frequently applied for symptomatic therapies in order to slow the neurodegenerative process [2]. However, despite huge contributions to clinical treatment, the efficiency was not satisfactory due to the various adverse effects [3]. So far, the clinical diagnosis of PD mainly depends on histopathology, which in turn needs invasive surgical brain biopsy and clinical manifestations using Unified Parkinson’s Disease Rating Scale (UPDRS)-V and UPDRS-III scores [4,5]. These two golden criteria are subjective, invasive and limited, which will seriously interfere with the accuracy of early PD detection. Thus, it is necessary to identify crucial biomarkers for the early diagnosis of PD.

MicroRNAs (miRNAs) are a group of small, non-coding and endogenous RNAs that regulate gene expression by binding to complementary sequences in post-transcriptional processes [6]. As reported, a number of miRNAs participated in different cellular processes, including proliferation, apoptosis and differentiation so as to regulate pathogenesis resulting in the development of various diseases [7,8,9]. The miRNA profiles have documented that several miRNAs were aberrantly expressed in serum samples obtained from PD patients, such as miR-29a-3p, miR-30a-5p, miR-30b-5p, miR-103a-3p, miR-153, miR309-3p and so on [10,11,12]. In a previous study, the expressions of miR-132-3p and miR-146a-5p generally decreased in PD patients compared to the unaffected controls [3,13], suggesting that these two miRNAs may be good candidates for detecting PD progression. Also, several miRNAs were identified as promising biomarkers for PD [14,15]. However, the clinical values of miR-132-3p and miR-146a-5p concerning the early diagnosis of PD still remained to be elucidated.

According to the previous studies [16,17,18,19], PD patients were divided into five different stages; and based on the severity of symptoms, they were divided into mild PD, moderate PD and severe PD. Mild PD was described as speech abnormalities, rigidity of the muscles in the trunk, rigidity, loss of facial expression or rigidity. Moderate PD was described as loss of balance and slowness of movement. Severe PD was described as severely disabling, such as delusions, hallucinations, unable to rise, unable to walk or freeze or stumble when walking. The aim of the present study was to find out a novel adjuvant method to help improve the accuracy of early diagnosis of PD. Hence, based on the previous study, we divided the PD patients into normal PD and severe PD to discriminate the two different severities of PD.

In the present study, we focused on miR-132-3p and miR-146a-5p to better investigate and understand their potential clinical values as PD biomarkers.

## 2 Materials and methods

### 2.1 Patients

A total of 82 patients with PD and 44 healthy volunteers were recruited from the Fourth Affiliated People’s Hospital of Jiangsu University between June 2015 and October 2017 (Table 1). The clinical diagnosis of PD was based on the criteria of Part V of the UPDRS (UPDRS-V; the modified Hoehn and Yahr staging of PD), Part III of the UPDRS (UPDRS-III) and magnetic resonance imaging.

**Table 1:** Clinical data of PD patients and healthy volunteers

Variables	PD (*n* = 82)	Healthy (*n* = 44)	*P* value
Age (years)	68.53 ± 7.53	66.24 ± 8.62	0.1217
Gender			
Male	52 (63.3%)	27 (61.4%)	0.7816
Female	30 (36.7%)	17 (38.6%)	
Family PD history	30 (36.6%)	2 (4.5%)	*P* < 0.0001
Smoking	11 (12.5%)	6 (13.6%)	0.8174
Alcohol abuse	15 (18.3%)	8 (18.2%)	0.9854
Diabetes	22 (26.8%)	13 (15.9%)	0.0600
Hypertension	33 (40.2%)	20 (45.5%)	0.4489
MMSE scores	28.2 ± 2.3	27.5 ± 3.2	0.1595
miR-132-3p (fold)	0.67 ± 0.04	0.99 ± 0.06	*P* < 0.0001
miR-146a-5p (fold)	0.66 ± 0.04	1.02 ± 0.06	*P* < 0.0001

PD, Parkinson’s disease; Healthy, healthy volunteers; MMSE, Mini-Mental State Examination. Data in age, MMSE scores, miR-132-3p and miR-146a-5p expression were analyzed by chi-square test, while other variables were analyzed using ANOVA followed by Bonferroni’s *post hoc* test.

Healthy controls fasted for 12 h before serum samples were taken, while PD patients were forbidden to take any anti-parkinsonian medications and fasted for 12 h before sample collection. Serum samples (5 mL) were taken and preserved in tubes without anticoagulant at room temperature following the protocols from Parkinson Progression Marker Initiative. Then the tubes were centrifuged for 15 min at 1,900 × *g* at 4°C. After centrifugation, all samples were immediately stored at −80°C until the subsequent analysis.


**Informed consent:** Informed consent has been obtained from all individuals included in this study.
**Ethical approval:** The research related to human use has been complied with all the relevant national regulations, institutional policies and in accordance with the tenets of the Helsinki Declaration, and has been approved by the Ethics Committee of the Fourth Affiliated People’s Hospital of Jiangsu University.

### 2.2 Reverse transcription-quantitative polymerase chain reaction (RT-qPCR) assay

Total RNA was isolated and extracted from serum samples using TRIzol reagent (Invitrogen, Thermo Fisher Scientific, Inc., USA) by following the instruction. Complementary DNA was synthesized using the SuperScript III First-Strand Synthesis kit (Invitrogen, Thermo Fisher Scientific, Inc., USA). The amplification with specific primers of miR-132-3p and miR-146a-5p was conducted using FastStart Essential DNA Green Master (Roche, Mannheim, Germany) on ProFlex™ PCR machine according to the protocol. The PCR thermal cycling conditions were as follows: denaturation at 96°C for 5 min, followed by 40 cycles of 96°C for 5 s and 62°C for 10 s. The U6 served as an endogenous reference gene to normalize the miRNAs, and the relative expressions of miRNAs were calculated using the 2^−ΔΔCt^ method. All the procedures were conducted in triplicate. The primers were as follows: miR-132-3p forward 5′-GCGCGCGTAACAGTCTACAGG-3′ and reverse 5′-GTCGTATCCAGTGCAGGGTCC-3′; miR-146a-5p forward 5′-CGAGTCCAGTTTTCCCAGGA′ and reverse 5′-GTCGTATCCAGTGCAGGG-3′; U6 forward 5′-CTCGCTTCGGCAGCACATATACT-3′ and reverse 5′-CGCTTCACGAATTTGCGTGT-3′.

### 2.3 Statistical analysis

Statistical analysis was carried out using SPSS version 20.0 software (IBM, USA). The chi-square test and ANOVA followed by Bonferroni’s *post hoc* test were used to distinguish between miR-132-3p and miR-146a-5p in patients with PD and healthy volunteers. Spearman’s rank correlation analysis was performed to assess the association between aberrant miRNAs and Braak staging, UPDRS-V, and UPDRS-III. Receiver–operating characteristic (ROC) curves were constructed and area under the curve (AUC) values were detected to identify the diagnostic values of miR-132-3p and miR-146a-5p for PD. Differences in all data were expressed as mean ± standard deviation (SD), and *P* < 0.05 was considered to be statistically significant.

## 3 Results

### 3.1 Clinical data

As demonstrated in Table 1, the clinical data of 82 PD patients and 44 healthy controls were compared. No differences were observed in age, gender, smoking, alcohol abuse, diabetes or hypertension. However, remarkable differences were detected in family PD history (*P* < 0.01), miR-132-3p (*P* < 0.01) and miR-146a-5p expression (*P* < 0.01) between PD patients and healthy volunteers.

### 3.2 The comparison of clinical data between groups divided based on PD severity

As described in Section 1, the PD patients were divided into normal PD and severe PD. As demonstrated in Table 2, no significant differences were observed in age and gender between the two groups. However, remarkable differences were found in UPDRS-III score (*P* = 0.0044), UPDRS-V score (*P* = 0.0006), Braak staging (*P* = 0.0006), miR-132-3p expression (*P* < 0.0001) and miR-146a-5p expression (*P* < 0.0001) between severe and normal PD groups.

**Table 2:** Different groups of PD patients divided by the severity of PD

Variables	PD (*n* = 82)	Severe (*n* = 34)	Normal PD (*n* = 48)	*P* value
UPDRS-III				
<10	12	3 (8.82%)	9 (18.75%)	—
10–30	34	7 (20.59%)	27 (56.25%)	
30–50	21	16 (41.06%)	5 (10.42%)	
>50	15	8 (23.53%)	7 (14.58%)	
UPDRS-V				
I	21	4 (11.76%)	17 (35.42%)	—
II–III	50	22 (64.71%)	28 (58.33%)	
IV–V	7	5 (14.71%)	2 (4.17%)	
>V	4	3 (8.82%)	1 (2.08%)	
Braak staging				
I–II	20	3 (8.82%)	17 (35.42%)	—
III–IV	49	23 (67.65%)	26 (54.17%)	
V–VI	13	8 (23.53%)	5 (10.42%)	
Age (years)	68.53 ± 7.53	67.32 ± 7.68	68.81 ± 7.04	0.3660
Gender				
Male	52	23	29	0.5126
Female	30	11	19	
Disease severity				
UPDRS-III scores	29.77 ± 10.63	35.68 ± 10.52	25.58 ± 11.07	0.0044
UPDRS-V scores	2.47 ± 0.64	3.03 ± 0.68	2.06 ± 0.56	0.0006
Braak staging	3.23 ± 0.50	3.82 ± 0.48	2.75 ± 0.51	0.0006
miR-132-3p (fold)	0.67 ± 0.07	0.52 ± 0.05	0.78 ± 0.09	*P* < 0.0001
miR-146a-5p (fold)	0.66 ± 0.06	0.57 ± 0.04	0.73 ± 0.07	*P* < 0.0001

Severe, severe PD patients; PD, Parkinson’s disease; —, not applicable; UPDRS, Unified Parkinson’s Disease Rating Scale; Data in age, disease severity, miR-132-3p and miR-146a-5p expression were analyzed with ANOVA followed by Bonferroni’s *post hoc* test, while other variables were analyzed by chi-square test.

### 3.3 Expression levels of miR-132-3p and miR-146a-5p in serum samples of PD patients and healthy volunteers

**Figure 1:**
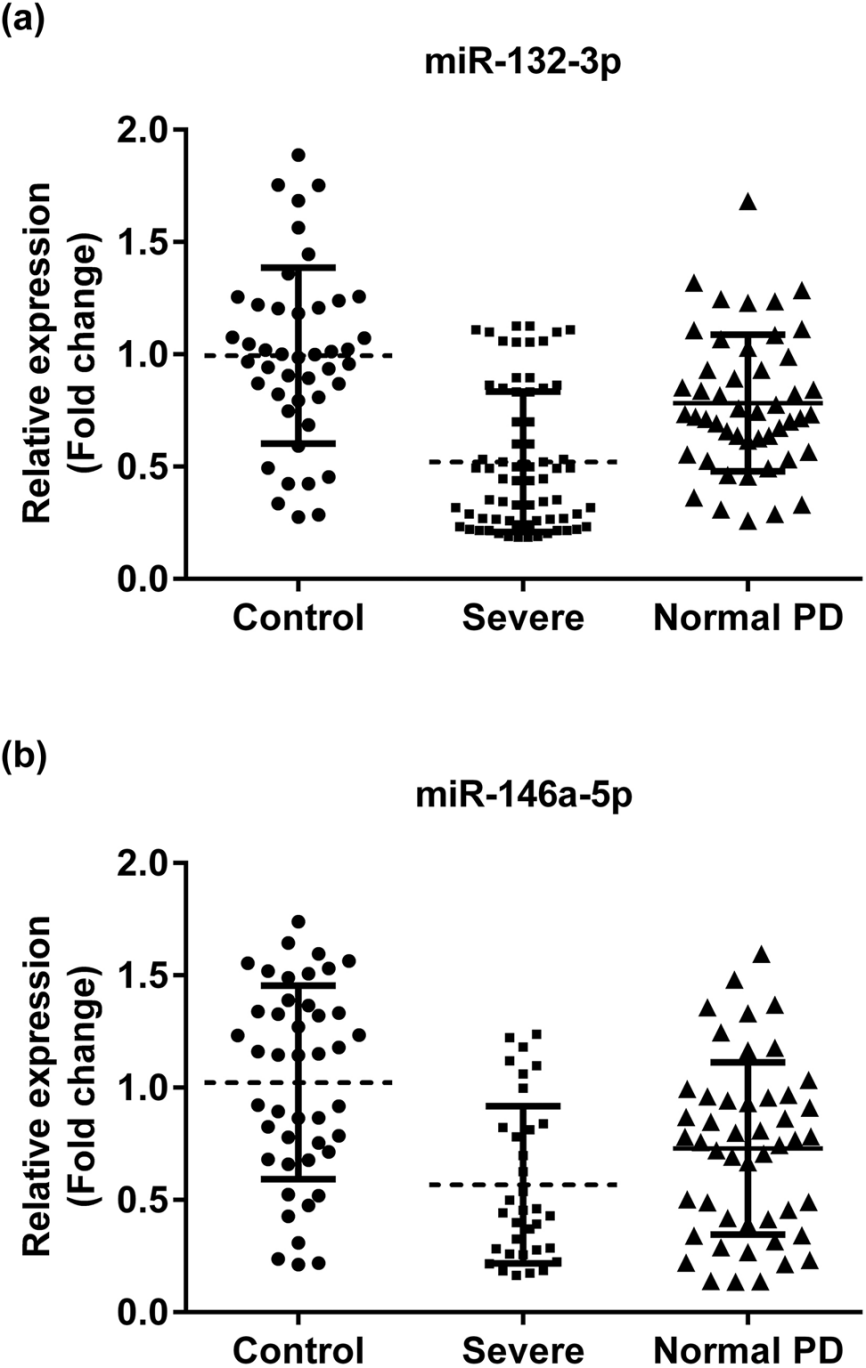
The expressions of miR-132-3p (a) and miR-146a-5p (b) in serum samples of 82 PD patients and 44 healthy volunteers using RT-qPCR analysis. **P < 0.01, PD vs controls. PD, Parkinson’s disease; severe, severe PD patients; control, healthy volunteers.

As demonstrated in Figure 1a and b, the expression levels of miR-132-3p and miR-146a-5p significantly decreased in the serum samples of PD patients (*P* < 0.01), as compared with those in healthy controls. Furthermore, compared with normal PD patients, the expressions of miR-132-3p and miR-146a-5p were dramatically decreased in severe PD groups (*P* < 0.01).

### 3.4 Correlation analysis of miR-132-3p and miR-146a-5p with Braak stage, UPDRS-V and UPDRS-III scores at admission

**Figure 2:**
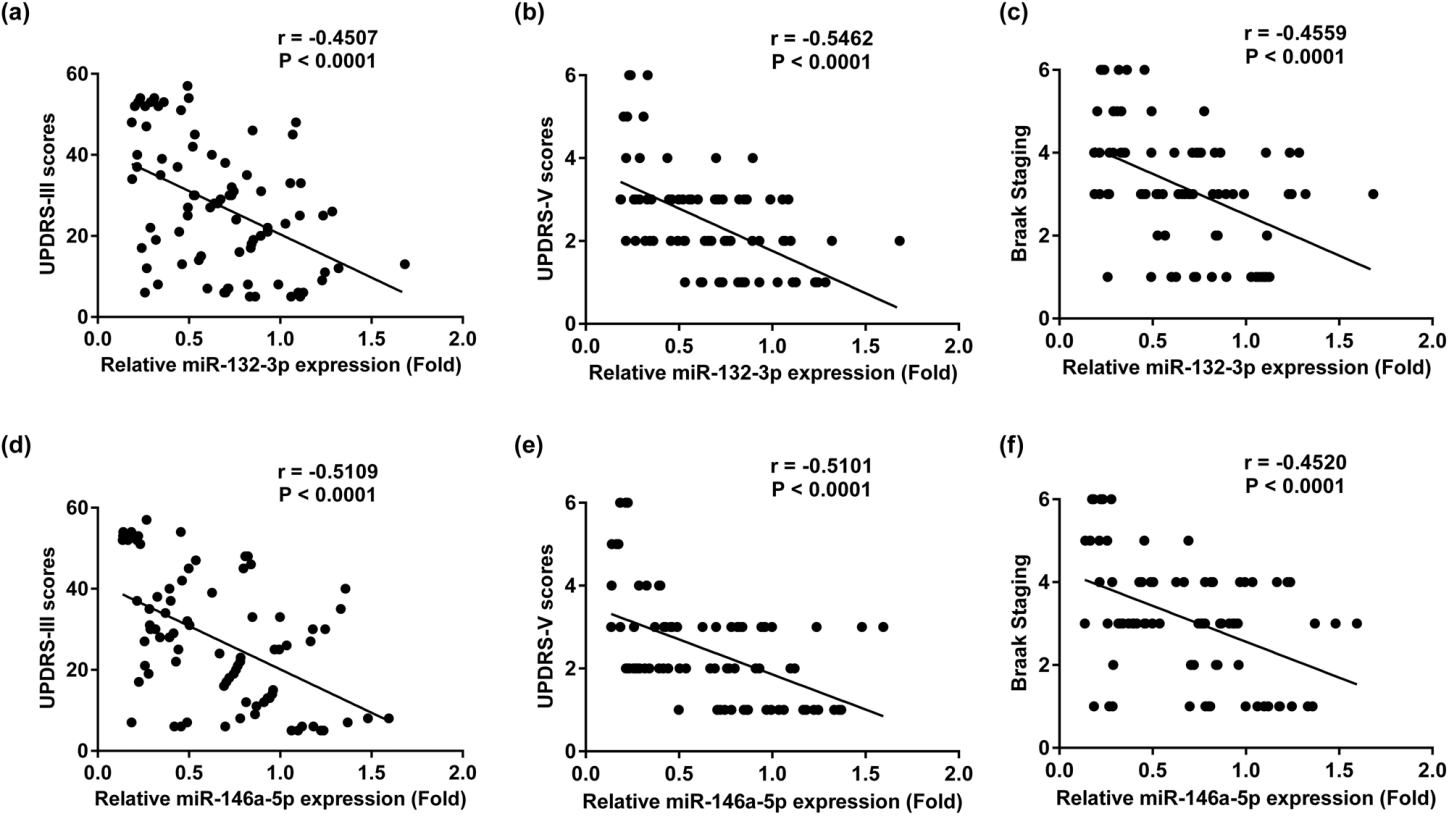
miR-132-3p and miR-146a-5p expressions were correlated with UPDRS-III, UPDRS-V scores and Braak staging in PD patients. (a) Serum miR-132-3p was negatively correlated with the UPDRS-III scores in PD patients. (b) Serum miR-132-3p was negatively correlated with UPDRS-V scores in PD patients. (c) Serum miR-132-3p was negatively correlated with Braak staging in PD patients. (d) Serum miR-146a-5p was negatively correlated with the UPDRS-III scores in PD patients. (e) Serum miR-146a-5p was negatively correlated with UPDRS-V scores in PD patients. (f) Serum miR-146a-5p was negatively correlated with Braak staging in PD patients.

Correlation analysis was performed using Pearson’s correlation analysis. As demonstrated in Figure 2a–c, the expression of miR-132-3p was negatively related to UPDRS-III (*r* = −0.45, *P* < 0.0001), UPDRS-V (*r* = −0.55, *P* < 0.0001) and Braak staging (*r* = −0.46, *P* < 0.0001). Meanwhile, the results were similar with regard to the expression of miR-146a-5p (*r* = − 0.51, *P* < 0.0001; *r* = −0.51, *P* < 0.0001; *r* = −0.45, *P* < 0.0001, respectively; Figure 2d–f).

### 3.5 ROC curve analysis

**Figure 3:**
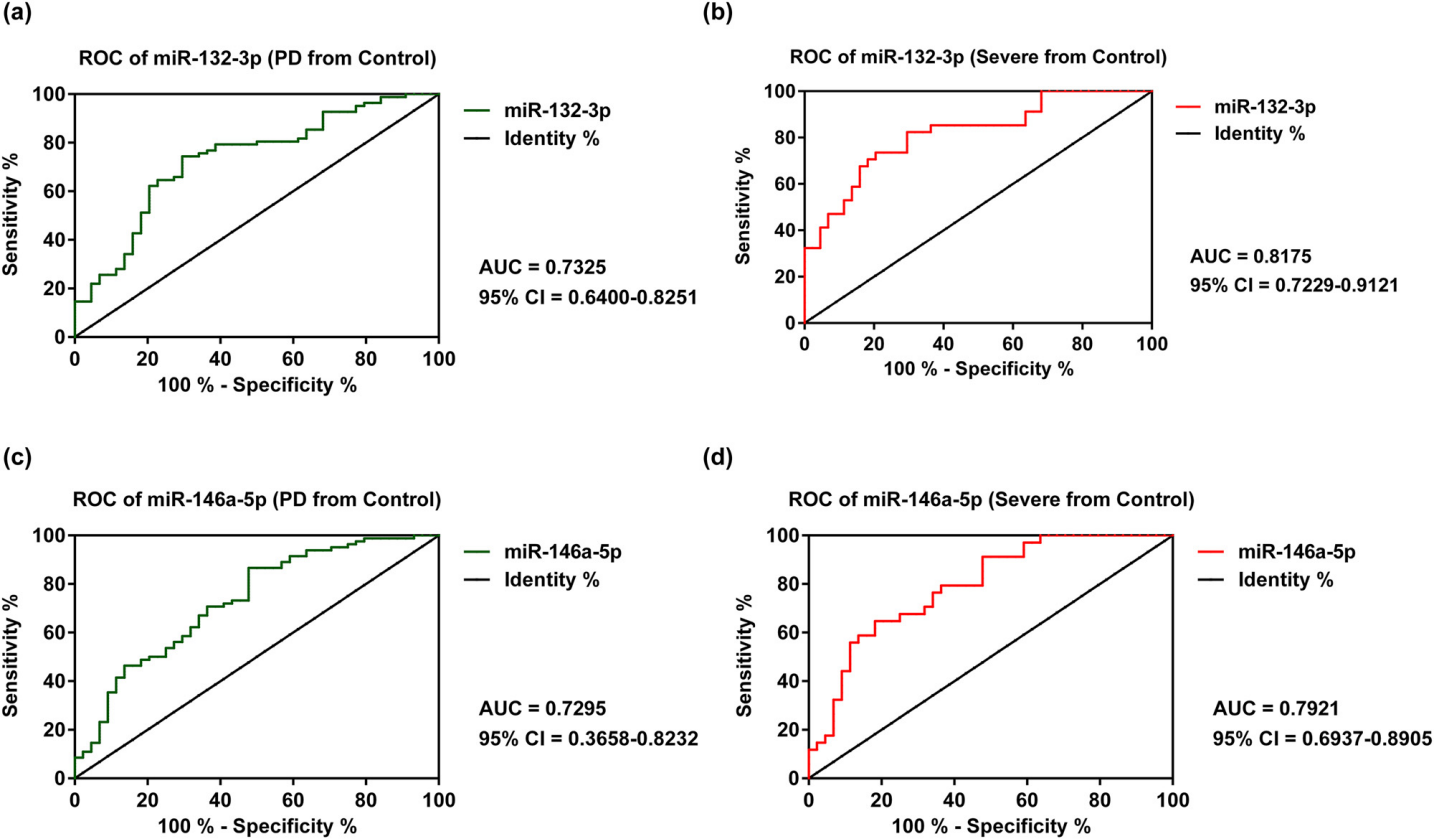
The ROC analysis of miR-132-3p (a and b) and miR-146a-5p (c and d) in discriminating PD cases from healthy controls and severe PD patients from normal PD patients.

The ROC curve analysis was carried out so as to measure the diagnostic value of miR-132-3p and miR-146a-5p for PD. As demonstrated in Figure 3a and c, the AUC of miR-132-3p and miR-146a-5p in discriminating PD from healthy controls was 0.7325 (95% CI = 0.6400–0.8251) and 0.7295 (95% CI = 0.3658–0.8232), respectively. Meanwhile, the AUC of miR-132-3p and miR-146a-5p in discriminating severe PD from normal PD patients was 0.8175 (95% CI = 0.7229–0.9121) and 0.7921 (95% CI = 0.6937–0.8905), respectively (Figure 3b and d).

## 4 Discussion

To date, neurological detection and neuroimaging are the basic and golden standards for physicians in the diagnosis of PD. However, both these criteria lack sensitivity and are subjective. Therefore, there is an urgent need to discover non-invasive, sensitive and accurate indicators for PD.

To the best of our knowledge, certain miRNAs were reported to be dysregulated in PD patients and may serve as promising biomarkers for PD diagnosis [20,21]. A previous study [3] has demonstrated that miR-4639 served as a potential biomarker for PD by regulating DJ-1 expression; meanwhile dysregulation of miR-4639 was correlated with UPDRS-I, UPDRS-II and UPDRS-III as well. Moreover, another group also illustrated that miR-132-3p and miR-146a-5p were abnormally expressed in neurodegenerative diseases such as PD [13]. However, to our knowledge, no reports have elucidated their potential role and mechanism in PD.

Both miR-132-3p and miR-146a-5p were dysregulated in various solid malignancies and also involved in human cancer and inflammation-related diseases [22,23,24,25]. In the present study, we found that miR-132-3p and miR-146-5p expressions were significantly decreased in PD patients and further decreased in severe PD patients compared with healthy participants and normal PD patients. Previously, serum miR-132-3p and miR-146-5p were reported to be decreased in PD patients, which is consistent with our study [13,26]. However, our report further determined their clinical values concerning clinical diagnosis. Braak staging system classifies the degree of pathology in PD and is widely used in clinical diagnosis and research. This system is normally divided into six different stages, i.e., stages 1–6, characterized by abnormal pathology, in particular neurological structures. UPDRS-III and UPDRS-V scores were the golden criteria for clinical diagnosis of PD. UPDRS-III is clinician-score monitored motor evaluation, while UPDRS-V is Hoehn and Yahr staging of PD severity. As shown in our study, serum miR-132-3p and miR-146a-5p have strongly negative correlation with Braak staging, UPDRS-III and UPDRS-V scores. More importantly, significant differences were observed among Braak staging, UPDRS-III and UPDRS-V scores between severe and normal PD patients.

The AUC results of miR-132-3p and miR-146a-5p in discriminating PD from healthy controls were 0.7325 and 0.7295, respectively. Moreover, the AUC results of ROC miR-132-3p and miR-146a-5p in discriminating severe PD patients from normal PD patients were 0.8175 and 0.7921, respectively. Taken together, the decreased miR-132-3p and miR-146a-5p may indicate the severity of PD patients, which help in contributing to clinical diagnosis. The earlier the diagnosis, the more effective the treatment will alleviate the symptoms.

However, the present study still has some limitations. The samples recruited in our study are relatively small, which results in limited accuracy. Moreover, the samples were recruited only in China, so it needs to be confirmed whether the results are applicable worldwide. Finally, a 3-year or 5-year follow-up analysis needs to be performed in the future in order to evaluate the prognosis values of miR-132-3p and miR-146a-5p in PD.

Our findings elucidate the clinical potentials of serum miR-132-3p and miR-146a-5p for evaluating the diagnosis for PD patients.
